# Attitude, Perceived Barriers, and Challenges Toward Implementing Resource-Appropriate Guidelines for Hematologic Malignancies: Physicians' Survey in Ethiopia

**DOI:** 10.1200/GO.23.00104

**Published:** 2023-10-05

**Authors:** Obsie T. Baissa, Workagegnehu Hailu, Fisihatsion Tadesse, Abdulaziz Abubeker, Munir Awol Aman, Diriba Fufa, Ora Paltiel

**Affiliations:** ^1^ Faculty of Medicine, Braun School of Public Health Community Medicine, Hadassah Medical Organization, Hebrew University of Jerusalem, Jerusalem, Israel; ^2^ Department of Medicine, College of Medicine and Health Science, University of Gondar, Gondar, Ethiopia; ^3^ Department of Medicine, College of Health Sciences, Addis Ababa University, Ababa, Ethiopia; ^4^ Department of Oncology, College of Health Sciences, Addis Ababa University, Ababa, Ethiopia; ^5^ Department of Pediatrics, Institute of Health Science, Jimma University, Jimma, Ethiopia

## Abstract

**PURPOSE:**

Cancer care in low-income countries poses formidable challenges. Care may be facilitated by resource-adapted guidelines, such as the National Comprehensive Cancer Network (NCCN) harmonized guidelines for sub-Saharan Africa (NCCN-HG). Understanding physicians' attitudes and knowledge toward guidelines, as well as patient- and resource-related barriers, is essential for promoting their effective implementation.

**METHODS:**

We conducted an online survey among oncologists, hematologists, internists, residents/fellows, and generalists treating hematologic malignancies in Ethiopia. We assessed attitudes toward the use of guidelines, institutional capacity, and barriers/determinants to effective care.

**RESULTS:**

Among the 47 physicians completing the survey (representing 64% of Ethiopian professionals treating hematologic malignancies), the majority (85%) reported using guidelines; however, only 22.7% (n = 10) used the NCCN-HG. While overall attitudes toward guidelines were favorable, 57.8% of physicians familiar with the NCCN-HG were either undecided or believed that it lowers the standard of care. Perceived lack of institutional regulation was negatively associated with guideline use (*B* = −3.23; *P* = .004). Lack of diagnostic facilities including immunohistochemistry and flow cytometry, supportive care, and poor utilization of guidelines were reported to be determinants of poor patient outcome. Regarding patient factors, 57.4% respondents identified treatment abandonment as an important contributor to poor outcome. Availability of chemotherapy/radiotherapy (89.4%), financial status (85.1%), distance from the hospital (74.5%), and harvest season (65%) had major influences on treatment decisions. Over 80% reported that targeted therapies were unavailable or rarely available.

**CONCLUSION:**

Awareness and usage of the NCCN-HG are limited among Ethiopian physicians. Lack of facilities, therapies, and regulation, in addition to patient-related factors, was identified as barriers to guideline adherence and determinants of poor outcome.

## Teaser

Limited awareness of harmonized guidelines among physicians while capacity is below par even for the basic strata.

## BACKGROUND

Clinical guidelines, by setting standards, have been shown to be effective in improving cancer care and quality of service in high-income countries.^1,2^ Resource-stratified guidelines were first introduced in 2005 by the breast heath global initiative and later adopted by ASCO and other oncology societies as evidence-based, economically feasible, four-tier resource-adapted practice guidelines for resource-limited settings.^1,3^ Recently, the African cancer coalition in collaboration with the National Comprehensive Cancer Network (NCCN) and ASCO produced harmonized, resource-integrated guidelines for 46 cancers including hematologic malignancies.^4^ These guidelines were designed by experts from the sub-Saharan Africa (SSA) region by prioritizing essential resources and services for a sustainable cancer care model across resource levels.^5^ The parent NCCN guideline designed for high-income settings was used as a reference and then stratified across four resource levels from the highest-cost recommendation to the minimum acceptable standards of care.^5^ Currently, 13 countries from SSA, including Ethiopia, have endorsed the NCCN harmonized guidelines for SSA (from here on called harmonized guidelines) to standardize cancer care delivery.^5^ However, feasibility and implementation strategies for resource-stratified cancer guidelines in African settings have not been fully explored.^6^ Only one study from the region explored the topic and reported that most oncologists use guidelines designed for high-income countries and further identified lack of proper infrastructure and complexity of guidelines as barriers to their implementation.^6^ In summary, there is a paucity of data on physicians' attitude, perceived barriers, and facilitators for improving guideline implementation in SSA.^6^ Therefore, we performed a cross-sectional online survey aiming to assess usage, attitude, perceived barriers, and facilitators, as well as the existing institutional capacity for implementing harmonized guidelines for hematologic malignancies in Ethiopia, to provide evidence for designing a tailored implementation strategy for the NCCN harmonized guideline in Ethiopia.

**CONTEXT**

**Key Objective**
To explore the facilitators, barriers, and challenges in implementing the recently endorsed harmonized guidelines for care of hematologic malignancies in Ethiopia from the physicians' perspective.
**Knowledge Generated**
While the overall attitude about guidelines in Ethiopia is favorable, surveyed physicians showed limited awareness and usage of harmonized guidelines. Moreover, most physicians believe that the current level of care offered is suboptimal and the current institutional capacity does not even meet the diagnostic and therapeutic requirements of the basic strata of the harmonized guidelines. Respondents voiced concern about providing a compromised standard of care to their patients.
**Relevance**
Our findings present valuable input to designing a tailored implementation strategy use of guidelines in Ethiopia and provide insights into the challenges of offering care for hematologic cancers in resource-poor countries, in general.

## METHODS

### Design Setting and Participants

A cross-sectional study design was implemented. We recruited oncologists, hematologists, residents in training, internists, and general practitioners, directly involved in the management of hematologic malignancies from three different referral/teaching hospitals in Ethiopia from July 2021 to March 2022. Physicians who did not treat patients with hematologic malignancies in the past 5 years were excluded from the study.

A convenience sampling method was used. Major teaching/referral hospitals in Ethiopia that treat hematologic malignancies were listed from the Ministry of Health website. Department heads at these centers (Addis Ababa University, Jimma University, University of Gondar) were contacted to facilitate recruitment of staff by forwarding the survey link to physicians who were practicing at hematology/oncology units in each hospital. We could not contact one major center because of an ongoing war. According to the information provided from departments, the target population was 80 physicians—60 physicians from Addis Ababa, six physicians each from the University of Gondar and Jimma University, and around 10 physicians in other referral hospitals. The questionnaire was administered online via the Google survey platform. The questionnaire had three sections and a total of 23 questions. To limit multiple participation, the survey was sent to a specific group of physicians via e-mail and sign-in was required to limit multiple entries (Data Supplement).

### Sample Size

With an estimated 80 physicians involved in the care of patients with hematologic malignancies in Ethiopia and 50% assumption of knowledge on NCCN harmonized guidelines, a minimum sample size of 44 provides 95% CI at a 10% margin of error.

### Study Design

The survey gathered information on the demographic and professional characteristics of physicians treating hematologic malignancies in Ethiopia. Survey questions also addressed usage, attitude, perceived barriers, facilitators, and infrastructural challenges for guideline adherence. Physicians' attitude toward practice guidelines was assessed via a tool adopted from a validated questionnaire by Tunis et al.^7^ Barriers toward clinical guidelines were evaluated using a framework elaborated by Cabana et al.^8^ Similarly, logistics, practice, and patient-related barriers were explored using the WHO Cancer early diagnosis guide to improve access to treatment targets as a reference.^9^ Current institutional capacity was evaluated through a 3-point scale (conditions we can deal with adequately, conditions that we offer treatment for but may not be optimal in 2021 but of proven efficacy, and conditions for which we cannot offer adequate treatment) adopted from a review by Luzzatto et al.^10^ Finally, the questionnaire was pretested/pilot tested via recruitment of 10 physicians from SSA studying for master's degrees at Hebrew University.

### Statistical Analysis

Statistical analysis was performed using IBM SPSS statistics version 26 (IBM Corporation, Armonk, NY). Physicians' characteristics and responses were summarized using frequencies and percentages. Baseline characteristics of the respondents were compared with the expected distribution and characteristics of the target physicians to assess for nonresponse bias. Statistical significance of associations between physician characteristics and dichotomous question responses (eg, awareness, familiarity) was analyzed using the chi-square test. Internal consistency between the attitude scales was measured using Cronbach's alpha. Favorable attitude characteristics showed acceptable levels of reliability (a = 0.9). Responses to perceived barriers were grouped into five separate categories such as knowledge, attitude, setup-related factors, guideline-related factors, and regulations, and ordinal regression models were used to explore relationships between guideline use and those barriers. Statistical significance was defined with *P* values of < .05. Missing data analysis showed that values were missing at random.

### Ethical Clearance

Participants were informed about the research goals of the survey in writing. By voluntarily participating in the survey, informed consent was assumed, but not obtained in writing. No remuneration was provided. Ethical approval was obtained from the committee for ethics in research involving human participants at the Faculty of Medicine, Hebrew University, study No. 17062021.

## RESULTS

A total of 47 physicians completed the online survey, with a total response rate of 64% representing the majority of Ethiopian physicians treating hematologic malignancies. As shown in Table 1, hematologists and oncologists constituted 44.7% of the survey respondents. Residents and hematology fellows accounted for 40.4%. All the surveyed physicians were working at either a teaching/referral (91.5%) or general hospitals (8.5%). Most of the participants (70.2%) reported below 5 years of clinical experience. In addition, 83% of the respondents were based in the capital city, that is, Addis Ababa University, whereas the remainder were working at the University of Gondar or Jimma University. Only one third of the physicians reported treating more than 10 patients with hematologic malignancies per week.

**TABLE 1:**
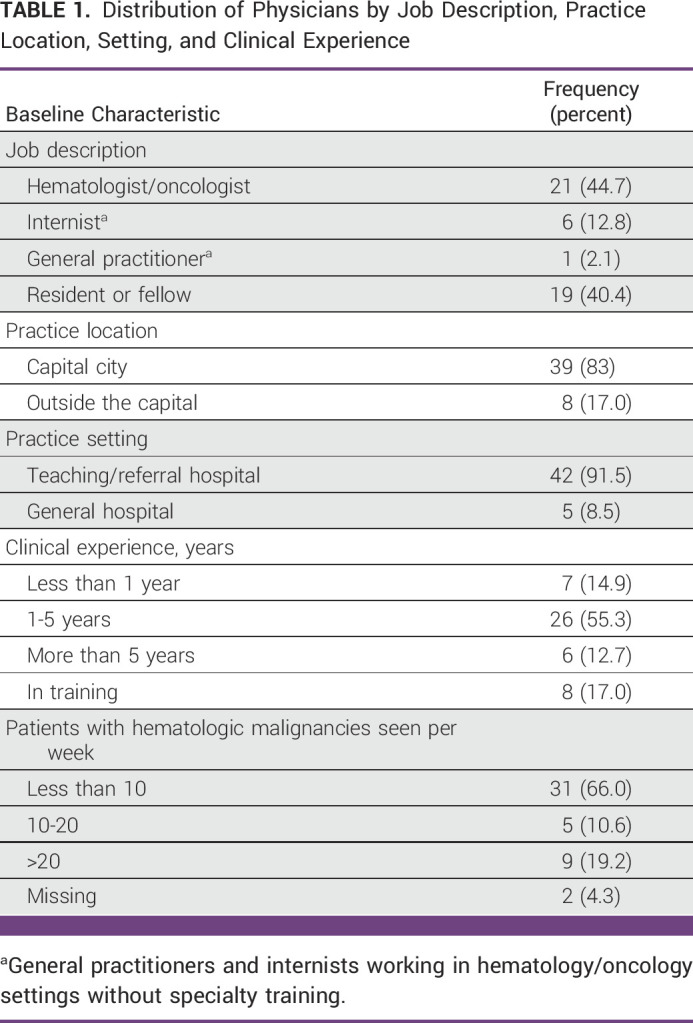
Distribution of Physicians by Job Description, Practice Location, Setting, and Clinical Experience

### Guideline Use

Over 85% of physicians reported using clinical guidelines often or very often while treating hematologic malignancies. Furthermore, 81.6% of the physicians considered guidelines as an extremely important (65.3%) or important (16.3%) tool in deciding a course of therapy. Over three fourth of physicians reported using the NCCN guidelines (81.8%), followed by European Society of Medical Oncology guidelines at 47.7% and ASCO guidelines at 38.6% in managing their patients. However, only 22.7% (n = 10) of physicians reported using the harmonized guideline for SSA.

Among the reasons for not using guidelines, 23.4% of physicians were not aware of any practical guideline that was useful for their setup. Moreover, 25.6% and 19.1% of physicians, respectively, reported lack of guideline-specific training and unfamiliarity with recommendations for poor adherence. In addition, 32.4% of physicians stated that the available guidelines were not convenient for their clinical setting, whereas 25.5% said that guidelines were not applicable for their patient population. A third (30.3%) of the respondents believed that the recommendations were too complex, whereas over half of the physicians (51.2%) responded that their setup did not have the technology to properly implement guidelines.

### NCCN Harmonized Guidelines

About half (48.9% [n = 23]) of physicians were aware of the harmonized guidelines for SSA. However, only 25.2% (n = 12) of physicians were familiar with its recommendations. Awareness and familiarity did not differ significantly by job description (*P* = .46). Among those who were familiar with the NCCN harmonized guidelines, 75% reported that harmonized guidelines were applicable to their patient population, whereas 66.7% and 75% of physicians believed that the harmonized guidelines could improve clinical outcomes, respectively. However, 57.8% of physicians were either undecided or believed that the harmonized guideline could lower the standard of care. Similarly, 41.7% of physicians did not believe that harmonized guidelines addressed the unique needs and comorbidities in their setting.

### Attitude Toward Clinical Guidelines

As described in Table 2, clinical guidelines were considered to be a good source (80.9% agreement), whereas 83% of physicians agreed that guidelines would likely improve quality of care for patients with hematologic malignancies. An even higher percentage (87.2%) of physicians agreed that guidelines are good educational tools. Only 63.8% of respondents were in agreement with the statement that clinical guidelines would reduce health care costs. In addition, 51.2% of the respondents had confidence in the guideline development process, whereas 57.4% believed that guidelines were evidence-based. By contrast, 17% of physicians reported that guidelines were developed by experts who understand little of daily clinical routine. Furthermore, <10% of physicians were in agreement with negative statements such as guidelines challenge autonomy or represented oversimplified medicine.

**TABLE 2:**
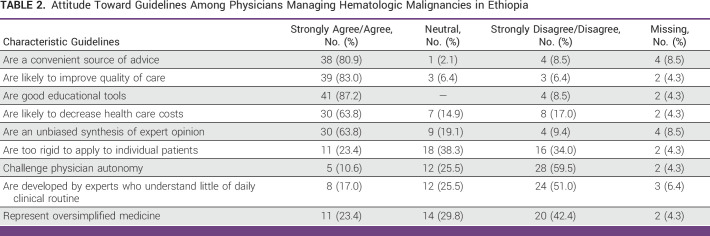
Attitude Toward Guidelines Among Physicians Managing Hematologic Malignancies in Ethiopia

### Perceived Barriers to Guideline Adherence

As shown in Table 3, 51% of physicians agree/strongly agree that perceived lack of regulation by the institution, that is, lack of the institutional mechanism to enforce compliance to guidelines or lack of institutional endorsement of a specific guideline, is a barrier to guideline adherence. In addition, in our regression model, perceived lack of regulation was negatively associated with guideline use (*B* = −3.23, *P* = .004). However, knowledge, attitude, setting, and guideline-related factors were not predictors of poor adherence.

**TABLE 3:**
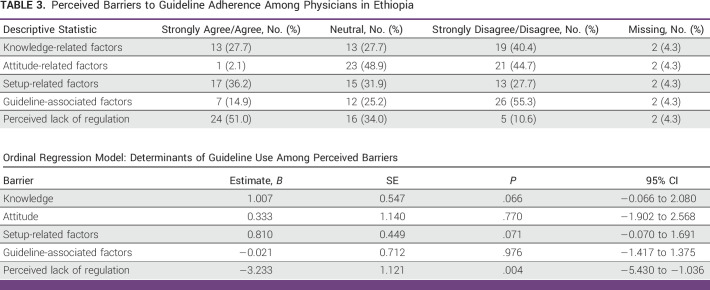
Perceived Barriers to Guideline Adherence Among Physicians in Ethiopia

### Factors Influencing Treatment Course

As shown in Table 4, on deciding a course of therapy for patients, almost all surveyed physicians reported that age and performance status influenced clinical decisions. Furthermore, HIV (76.6%) and other comorbidities such as tuberculosis and hepatitis B (76.6%) were also important in deciding on therapy. In addition, 89.4% of physicians reported that availability of chemotherapy/targeted therapy and radiotherapy also influenced their decision. Among nonclinical factors, 85.1% of physicians believed that the financial status of the patient was important in deciding a course of therapy. Furthermore, 74.5% of physicians reported that the need for the patient to travel to receive care influenced their decision on treatment modality. The patient's family status was also important (34.1%) and somehow important (31.9%) in decision making. In addition, two thirds of the physicians surveyed reported that time of the year such as harvest season was important in treatment decisions.

**TABLE 4:**
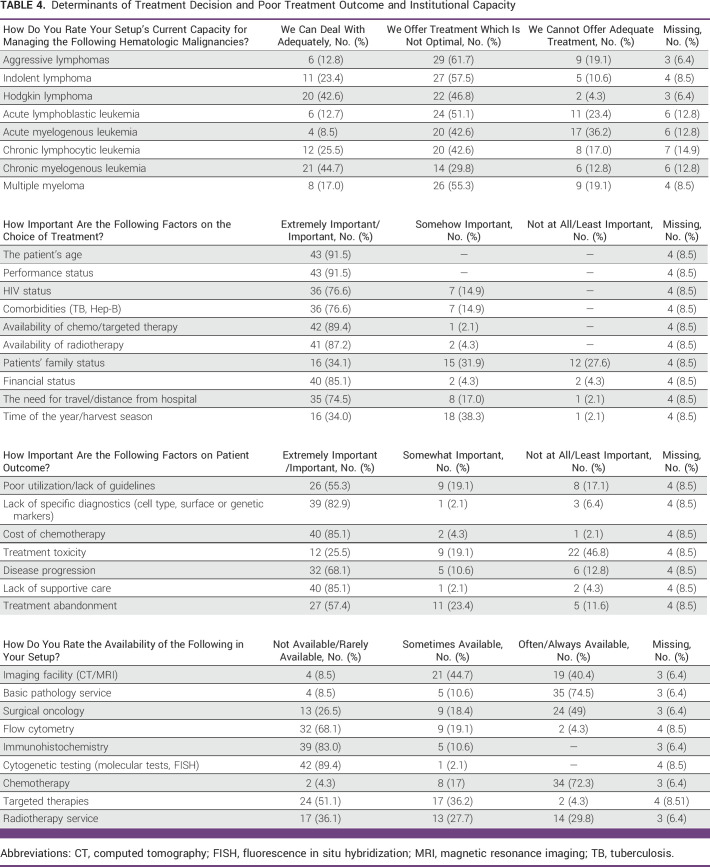
Determinants of Treatment Decision and Poor Treatment Outcome and Institutional Capacity

As also described in Table 4, 82.9% of physicians considered lack of specific diagnostic facilities as a main factor for poor treatment outcome. Meanwhile, 85.1% of physicians reported that lack of supportive care was a major cause of poor treatment outcome. Treatment abandonment was considered an important (57.4%) and somewhat important (23.4%) issue in the care of hematologic malignances in their setting. Meanwhile, about half of the surveyed physicians (55.3%) believed that poor utilization or lack of clinical guidelines contributed to poor outcome in their facility. Regarding therapy, two third of physicians surveyed considered disease progression as a major cause of death rather than treatment toxicity.

As shown in Table 4, most of the physicians believed that the treatment offered was not optimal as per the current standard of care but nevertheless proven to be effective. A third of the physicians (36%) believed that acute myeloid leukemia cannot be adequately managed in the current setting, whereas 41.5% believed that the treatment offered was not optimal but nevertheless proven to be effective. Almost two third of physicians (61.7%) reported that aggressive large-cell lymphoma management was suboptimal, whereas 12.8% believe that it can be managed adequately in the current setting. In the case of Hodgkin lymphoma, 42.6% believed that it can be managed adequately, whereas 46.8% of physicians believed that it was suboptimal. As further described in Table 4, 72.4% and 79.6% of physicians, respectively, reported that chemotherapy and basic pathologic services were often available. Meanwhile, 40.4% of physicians reported that imaging facilities such as magnetic resonance imaging and computed tomography scans were often available. By contrast, flow cytometry, immunohistochemistry, and molecular testing were not available or rarely available. Facilities such as radiotherapy services and targeted therapy were not available or rarely available.

### Optimism

Almost 62% of physicians were optimistic about the country's ability to provide curative care for hematologic malignancies in the next 10 years, whereas 20% of physicians were not optimistic about this prospect in the Ethiopian setting.

## DISCUSSION

In this study, we assessed guideline use, barriers, facilitators, and existing infrastructure for guideline implementations in the management of hematologic malignancies in Ethiopia from physicians' perspective. Our findings showed that most physicians used international guidelines designed for high-income countries, whereas less than a quarter was familiar with the harmonized guidelines. There was an overall favorable attitude toward guidelines; nevertheless, over half of the physicians who were familiar with the harmonized guidelines were concerned that these guidelines could lower quality of care. Furthermore, our findings identified several barriers to guideline adherence such as the setup, patient-related factors, lack of regulation, and knowledge-related factors. Most physicians believed that the care provided was suboptimal. Furthermore, basic essential cancer care modalities including basic pathology and chemotherapy were at times unavailable. A majority of physicians attributed poor treatment outcome to poor supportive care, inadequate facilities, treatment abandonment, and disease progression.

Our study showed that over 90% of physicians used clinical guidelines in the management of hematologic malignancies. However, over three fourth of physicians relied on international guidelines. By contrast, less than a quarter of the physicians were familiar with the harmonized guideline. This is consistent with a survey conducted among oncologists from Nigeria, which reported a 93% guideline usage.^6^ In addition, reliance on guidelines from high-income countries reported in our study was also observed in the Nigerian survey whereby 81% of the oncologists used NCCN and ASCO guidelines. Another survey that included physicians from low-middle-income countries outside of SSA also showed that international guidelines were frequently used for patient management despite the fact that the procedures and therapies proposed in these guidelines may not actually be available.^11^

Overall, about 75% of clinicians surveyed had a positive attitude toward clinical guidelines. However, over half of the respondents who were familiar with the harmonized guideline either were undecided or believed that the harmonized guidelines could lower standard of care. The favorable response toward guidelines is consistent with multiple studies that reported favorable attitude toward guidelines among physicians.^7,12,13^ Contrary to our findings, other studies have suggested that experts believe that resource-appropriate care improves treatment outcome among patients with cancer.^1,4,5^ However, our finding mirrors the views of the proponents of the social justice approach that calls for expanded resources, block bargaining, etc rather than modifying guidelines in low-resource settings.^14^

Our findings revealed that nonmedical factors such as financial status of the patient, distance from the hospital, and availability of resources had a major influence on physicians' decision making. This is consistent with studies from Ethiopia and Ghana, which showed that when faced with limitations, physicians tend to use criteria such as younger age and being an economic provider in a family to offer treatment.^15,16^ The timing of harvest season was also an important consideration, and this is relevant in a country where 80% of the population are farmers living in rural areas. In addition, practices such as trade-offs between patient health needs and family welfare and a tendency by physicians to decide against treatment to avoid financial calamity on the family were observed.^15-17^ Similarly, poor treatment outcomes and treatment abandonment go hand in hand with the financial status of the patient.^17^

The NCCN harmonized guideline uses resource stratification, with the basic resource strata being defined as the minimal standard of care to improve treatment outcomes.^1,3^ Our findings showed that the essential bare minimum for the management of hematologic malignancies was not available or rarely available. This highlights the dilemma of providing treatment, whereas the basic diagnostic essentials and therapeutics are not met.

The major strength of our study lies in the design whereby we used established frameworks and questionnaires to assess guideline use, attitude, and perceived barriers. In addition, we pretested our questionnaire and its application to the specific group. Second, we conducted a multicenter study across three institutions and expanded our inclusion criteria to include residents, internists, and general practitioners, which reflects the current oncology workforce in Ethiopia. Finally, our survey comprehensively assessed the overall cancer care landscape in Ethiopia from the physicians' perspective.

Our study also has some limitations. Most of the respondents were based in the capital and practice at teaching/referral hospitals, and hence, the generalizability of our results to other parts of the country and settings may be limited. However, our data reflect the existing reality where most of the specialized workforce and cancer care facilities in Ethiopia are based either in the capital or in big cities. Furthermore, since our assessment of guideline usage and attitude was based on self-report and the fact that significant proportion of our respondents were fellows/residents, response bias and a tendency to over-report good behavior cannot be ruled out. However, our result was comparable with similar studies that assessed guideline usage and attitude. Furthermore, the survey was conducted anonymously, reducing the risk of social desirability bias.^18^ Finally, given the 64% response rate in our survey, we cannot rule out selection bias and a tendency for participants with positive attitude toward guidelines. However, the overall distribution of baseline characteristics of the respondents was comparable with the expected distribution in the country.

In conclusion, awareness and usage of the harmonized guideline are limited, despite being endorsed by the Ethiopian Ministry of Health. Furthermore, the existing infrastructure does not meet the essential bare minimum even for the basic strata of the NCCN harmonized guidelines for SSA. In addition, most of the care offered was reported to be suboptimal by the treating physicians. Finally, despite the challenges, over half of the physicians were optimistic about the prospect of curative cancer care in the near future.

Designing a resource-tailored implementation strategy and prioritization of available resources to meet at least the basic standard of care stipulated in the NCCN harmonized guideline are vital to improve treatment outcomes among patients with hematologic malignancies in Ethiopia and in all low-income settings. Findings of this study could provide a valuable input to design a tailored implementation strategy for cancer treatment in Ethiopia specifically and SSA, in general.
